# De novo cancer incidence after kidney and liver transplantation: Results from a nationwide population based data

**DOI:** 10.1038/s41598-019-53163-9

**Published:** 2019-11-20

**Authors:** Boyoung Park, Junghyun Yoon, Dongho Choi, Han Joon Kim, Yun Kyung Jung, Oh Jung Kwon, Kyeong Geun Lee

**Affiliations:** 1 Department of Medicine, College of Medicine, Hanyang University, Seoul, South Korea; 2 Graduate School of Public Health, Hanyang University, Seoul, South Korea; 3 Department of Surgery, College of Medicine, Hanyang University, Seoul, South Korea; 4 Hanyang ICT fusion medical research center, Seoul, South Korea

**Keywords:** Cancer prevention, Risk factors

## Abstract

The cancer risk among solid organ transplantation recipients in East-Asia has been insufficiently studied. This study estimated *de novo* cancer incidence in kidney and liver recipients 2008–2015, compared with the general population in Korea using nationwide data. This is a retrospective cohort study using nationwide health insurance claims data. The study population was comprised of cancer-free 10,085 kidney recipients and 3,822 liver recipients. Standardized incidence ratio (SIR) of cancer using indirect standardization was calculated. Compared with the general population, the cancer risk increased by 3.19-fold in male and 2.56-fold in female kidney recipients. By cancer type, a notably increased SIR was observed for Kaposi sarcoma, renal cancer, skin cancer, and non-Hodgkin’s lymphoma in male and for bladder cancer, renal cancer, and non-Hodgkin’s lymphoma in female kidney recipients. In liver recipients, the SIR of all cancers was 3.43 in males and 2.30 in females. In male liver recipients, the SIRs for Kaposi sarcoma, non-Hodgkin’s lymphoma, myeloid leukemia, and skin cancer and in female recipients those for non-Hodgkin’s lymphoma and liver cancer were prominent. A greatly higher SIRs for overall cancer and non-Hodgkin’s lymphoma in kidney and liver recipients aged 0–19 were observed, compared with recipients in other age group. The incidence of *de novo* cancer in kidney and liver recipients was higher than the general population and common types were different. Strategies of cancer prevention and screening after kidney and liver transplantation should be developed in response to the incidence of common types of *de novo* cancers.

## Introduction

Solid organ transplantation is the lifesaving procedure for patients with end organ diseases^1^. Worldwide, the number of organ transplantation has increased from 19,864 in the year of 2000 to 102,664 in 2017 and kidney and liver transplantation accounts for about 90% of total transplantations^2^. The outcomes after transplantation have continuously improved with better grafting, and survival, followed by longevity of the transplant organs^3,4^ with the 10-year graft survival of 82% in kidney recipients^3^ and 5-year survival rate of 81.2% in patients with liver transplantation^4^. Thus, malignancies after transplantation have become an increasingly important issue in terms of morbidity and mortality. In kidney and liver transplant recipients, *de novo* malignancies shows more aggressive tendency^5^ and is the important cause of late complications and mortality^6,7^. Previous studies have shown that *de novo* cancer incidence is generally higher in solid organ recipients with standardized incidence ratio (SIR) two to six-fold for kidney recipients and two to four-fold in liver recipients^5,8–10^. In addition, the cancer recurrence rate increased by 1.6 times in these subjects, especially in kidney transplant recipients^11^.

It has been suggested that an increased cancer risk is largely attributed to immune deficiency due to immunosuppressive therapy after transplantation, considering similar pattern of cancer incidence between two groups of immunosuppressed patients – people with HIV/AIDS and transplant recipients^12^. Previous studies have shown that viral infection related cancer risk including non-Hodgkin’s lymphoma, Hodgkin’s lymphoma, Kaposi sarcoma, anogenital cancer, and liver cancer are increased in solid organ transplant recipients. In addition, other types of cancer unrelated to viral infection are increased^5,6,8–10,13–20^. Most of these studies have conducted in Western populations, predominantly in Caucasians, and studies in Asian countries were mostly focused on Hong Kong with transplant registry data^13^ and Taiwan where the National Health Insurance program and database has been established^14–17^.

In Korea, where the fractions of cancers attributable to infection is the most important cause among preventable risk factors (21.2% of all cancer incidence in the year of 2009)^21^, the cancer incidence pattern in solid organ recipients who had higher susceptibility to risk of infection related cancer, needs to be accessed. Therefore, we estimated the risk of cancer incidence in kidney and liver recipients from 2008 to 2015 compared with general population using nationwide population-based database.

## Methods

This is a retrospective cohort study using health insurance claims data provided by the Health Insurance Review & Assessment (HIRA) database. South Korea has a mandatory universal health coverage system and the National Health Insurance covers more than 98% of the population. HIRA system is responsible for medical claims, medical claims review, and healthcare resources management in Korea. The HIRA claims data contains about 46 million patients per year and include claims from almost 80,000 healthcare service providers across South Korea with patients’ diagnosis, treatment, procedures, surgical history, and prescription drugs^22^.

**Figure 1:**
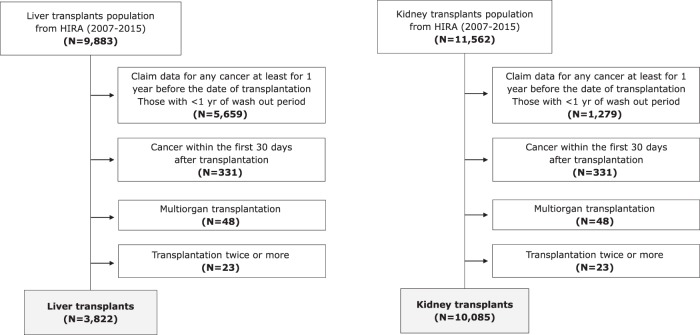
Flow diagram of the study population selection. The subjects were excluded consecutively. The excluded 6,061 liver recipients consisted of 5,659 people who had claim data for any cancer for <1 year before the date of transplantation, 370 who developed cancer within 30 days after transplantation, 78 who received multi-organ transplants, and 66 who received ≥2 transplants. The excluded 1,477 kidney recipients consisted of 1,279 people who had claim data for any cancer for <1 year before the date of transplantation, 38 who developed cancer within 30 days after transplantation, 78 who received multi-organ transplants, and 133 who received ≥2 transplants.

Based on the health insurance claims, data on patients who had received kidney or liver transplantation from January 2007 to December 2015 was extracted. The cancer incidence was identified as catastrophic illnesses in the National Health Insurance system and C code of International Classification of Diseases, 10th Revision (ICD-10). People with catastrophic illnesses are reimbursed for their co-payment to help patients who need costly treatment to receive necessary medical services. Among kidney or liver recipients between 2007 and 2015, we excluded subjects for the following criteria: (1) people who had claim data for any cancer for at least one year before the date of transplantation, (2) people who received transplantation in the year of 2007 (because we used the claim data from 2007, thus the claim data for cancer before transplantation date for the last one year could not be identified for them), (3) those who received multi-organ transplantation or transplantation twice or more, and (4) those who developed cancer within the thirty days after transplantation because they had a possibility have cancer before transplantation^14^. This study protocol was approved by the Institutional Review Board of the Hanyang University, Korea (Approval no: HYI-18-110). All procedures were performed in accordance with the Declaration of Helsinki 7^th^ version. This is a study for analysis of national health insurance database, thus informed consent was not obtained. Instead, all data was anonymized, thus we cannot access personal identifiable information. The flow of selection of study population was presented in the Fig. 1.

Cancers were first classified according to ICD-10 C codes including C00-C99, and then subdivided according to site of cancer. If a recipient was diagnosed with multiple cancers after transplantation, only the first cancer was considered. Cancer incidence in kidney or liver recipients was compared with the Korean general population between 2008 and 2015 from the Korea Central Cancer Registry, with SIR using indirect standardization. The equation of SIR is as follows:

$$Standardized\,incidence\,rate=\frac{observed\,number\,of\,de\,novo\,cancers}{Expected\,number\,of\,de\,novo\,cancers}$$

The expected number of total new cancers and site-specific cancer was calculated by multiplying age-specific cancer incidence rates in general population for 2008–2015 and the number of person-years of kidney or liver recipients. The 95% confidence intervals (95% CIs) for SIRs were calculated based on Poisson distribution. All analyses were performed separately by sex. In addition, age group at transplantation (0–39, 40–64, 65+) was also considered in subgroup analysis. SAS software version 9.4 (SAS Institute Inc., Cary, NC, USA) was used for all statistical analysis.

### Ethical approval and informed consent

This study protocol was approved by the Institutional Review Board of the Hanyang University, Korea (Approval no: HYI-18-110). All procedures were performed in accordance with the Declaration of Helsinki 7^th^ version. This is a study for analysis of national health insurance database, thus informed consent was not obtained. Instead, all data was anonymized, thus we cannot access personal identifiable information.

## Results

During 2007–2015, 11,562 kidney transplantation recipients and 9,883 liver transplantation recipients were identified. After exclusion of the relevant participants, the study population was comprised of 10,085 kidney transplantation recipients including 5,961 males and 4,124 females and 3,822 liver transplantation recipients including 2,329 males and 1,493 females (Fig. 1 and Table 1). The mean and median age at transplantation was 45.2 years and 50.0 years in kidney recipients and those were 46.0 years and 47.5 years in liver recipients. The total follow-up time was 36,234.8 years in kidney recipients and 14,622.1 years in liver recipients. The median follow-up time was 3.8 years for kidney recipients, including 2.9 years for *de novo* cancers; 1.9 years for hematopoietic cancer including non-Hodgkin’s lymphoma, leukemia, and lymphoid/hematopoietic cancers; and 3.0 years for patients with non- hematopoietic cancers. For liver recipients, the median follow-up time was 3.7 years, including 1.0 years for *de novo* cancers, 0.8 years for hematopoietic cancer, and 1.0 years for non- hematopoietic cancers.

**Table 1:** Characteristics of the kidney and liver transplant recipients included in the study.

	Kidney		Liver	
	N	%	N	%
Total	10,085	100.0	3,822	100.0
**Sex**				
Male	5,961	59.1	2,329	60.9
Female	4,124	40.9	1,493	39.1
**Age at transplant**				
0–9	37	0.6	121	7.5
10–19	165	2.8	31	2.1
20–29	479	8.0	55	3.2
30–39	1,035	17.4	205	8.7
40–49	1,624	27.2	711	26.2
50–59	1,875	31.5	926	37.6
60–69	691	11.6	266	13.8
70–	55	0.9	14	0.9
**Year of transplantation**				
2008	894	8.9	429	11.2
2009	962	9.5	432	11.3
2010	977	9.7	450	11.8
2011	1,354	13.4	478	12.5
2012	1,475	14.6	468	12.2
2013	1,458	14.5	475	12.4
2014	1,445	14.3	501	13.1
2015	1,520	15.1	589	15.4
Total person-years	36284.84		14622.08	

### Cancer incidence after kidney transplantation

In kidney transplantation recipients, 465 cancer cases developed including 289 cases in males and 176 cases in females (Table 2). The crude overall cancer incidence rates were 1368.5 per 100,000 person-years in males and 1160.4 per 100,000 person-years in females (data not shown). In males, renal cancer (n = 41), stomach cancer (n = 33), and cancer in thyroid and endocrine glands (n = 31) were most commonly developed and cancer in thyroid and endocrine glands (n = 41), breast cancer (n = 30), and renal cancer (n = 17) were the most commonly developed cancers in females.

**Table 2:** Standardized incidence ratio of different types of cancer in kidney and liver transplant recipients.

		Kidney recipients								Liver recipients							
		Male				Female				Male				Female			
Cancer site	ICD-10	O	E	SIR	95% CI	O	E	SIR	95% CI	O	E	SIR	95% CI	O	E	SIR	95% CI
All cancers	C00-C96	289	90.6	3.2*	2.8–3.6	176	68.8	2.6*	2.2–2.9	149	43.4	3.4*	2.9–4.0	64	27.8	2.3	1.7–2.9
Lip, Oral cavity and Pharynx	C00–C14	5	2.1	2.4	0.3–4.5	2	0.5	4.1	0.0–9.9	5	1.0	5.0	0.6–9.3	0	—	—	—
Stomach	C16	33	18.5	1.8*	1.2–2.4	15	5.1	2.9*	1.4–4.4	15	9.0	1.7	0.8–2.5	6	2.4	2.5	0.5–4.6
Colorectal	C18	6	7.3	0.8	0.2–1.5	4	3	1.4	0.0–2.7	10	3.5	2.8*	1.1–4.6	3	1.5	2.0	0.0–4.2
Liver	C22	23	12.6	1.8*	1.1–2.6	1	1.8	0.6	0.0–1.6	43	6.3	6.9*	4.8–8.9	11	1.0	11.3*	4.6–18.0
Other digestive organ	C15–C25^a^	14	13.1	1.1	0.5–1.6	4	4.2	0.9	0.0–1.9	10	6.3	1.6	0.6–2.6	2	2.2	0.9	0.0–2.2
Lung	C33–C34	24	9.7	2.5*	1.5–3.5	6	2.6	2.3	0.5–4.1	11	4.5	2.4	1.0–3.8	2	1.4	1.4	0.0–3.4
Other respiratory & intrathoracic	C30–C39^b^	5	1.4	3.5	0.4–6.6	1	0.2	4.5	0.0–13.3	0	—	—	—	0	—	—	—
Kaposi sarcoma	C46	9	0	379.8*	131.7–628.0	1	0	338.8	0.0–1002.7	1	0.0	93.4	0.0–276.5	0	—	—	—
Other bone, skin, and Soft tissue	C40-C49^c^	30	2.1	14.4*	9.2–19.5	5	1.1	4.5	0.6–8.5	7	1.0	7.2*	1.9–12.6	3	0.5	5.5	0.0–11.8
Breast	C50	0	—	—	—	30	14.3	2.1*	1.4–2.9	0	—	—	—	9	5.2	1.7	0.6–2.8
Female genital	C51-C58	0	—	—	—	17	6.5	2.6*	1.4–3.9	0	—	—	—	7	2.5	2.8	0.7–4.8
Male genital	C60–C63	14	4.9	2.9*	1.4–4.4	0	—	—	—	11	2.2	5.0*	2.1–8.0	0	—	—	—
Kidney	C64	41	2.8	14.6*	10.2–19.1	17	0.7	22.7*	11.9–33.6	3	1.4	2.2	0.0–4.7	1	0.3	2.9	0.0–8.5
Bladder	C67	6	2	3.1	0.6–5.5	6	0.2	28.4*	5.7–51.1	2	0.9	2.2	0.0–5.1	0	—	—	—
Other urinary tract	C64–C68^d^	0	—	—	—	1	0.1	11.2	0.0–33.1	0	—	—	—	0	—	—	—
Eye and Brain	C69–C72	3	0.9	3.5	0.0–7.5	1	0.5	2.1	0.0–6.3	1	0.4	2.5	0.0–7.4	0	—	—	—
Thyroid and Endocrine glands	C73–C75	31	7.5	4.1*	2.7–5.6	41	25.0	1.6*	1.1–2.1	2	3.4	0.6	0.0–1.4	8	8.8	0.9	0.3–1.6
non-Hodgkin’s lymphoma	C82-C86, C96	22	1.8	12.2*	7.1–17.3	13	0.8	16.1*	7.4–24.9	13	0.8	15.3*	7.0–23.7	10	0.4	27.0*	10.3–43.7
Leukemia	C92–C94	8	0.9	8.5*	2.6–14.4	1	0.5	2.1	0.0–6.2	6	0.4	14.1*	2.8–25.4	0	—	—	—
Lymphoid and Haematopoietic	C81–C96^e^	3	1.4	2.2	0.0–4.6	5	0.8	6.2	0.8–11.6	4	0.7	6.1	0.1–12.0	2	0.4	5.1	0.0–12.2

O: Observed number of cancer cases; E: Expected number of cases; SIR: Standardized incidence ratio; *significance level: <0.05

^a^*except C16, C18, C22;* ^b^*except C33,34;* ^c^*except C46;* ^d^*except C6*^*4*,^ *C67;* ^e^*except C82–86, C92–94, C96*

The SIR of all cancer development were 3.19 (95% CI = 2.82–3.55) in males and 2.56 (95% CI = 2.18–2.94) in females. The highest SIR was observed for Kaposi sarcoma development for both males and females but statistical significance was observed in only males (SIR = 379.80, 95% CI = 131.70–628.00 in males; SIR = 338.80, 95% CI = 0.00–1002.70 in females). In males, the SIR of renal, skin, and non-Hodgkin’s lymphoma were following (SIR = 14.63, 95% CI = 10.15–19.10; SIR = 14.35, 95% CI = 9.21–19.48; SIR = 12.16, 95% CI = 7.08–17.25). Additionally, the SIRs of stomach, liver, lung, male genital, thyroid and endocrine gland, and myeloid leukemia also increased. The SIRs for bladder cancer, renal cancer, and non-Hodgkin’s lymphoma were prominent with statistical significance (SIR = 28.39, 22.74, 16.12, respectively) and cancer of stomach, breast, female genital, and thyroid and endocrine glands also showed increased development in females, compared with general female population.

### Cancer incidence after liver transplantation

After transplantation, 213 cancer cases, including 149 males and 64 females, developed in liver recipients (Table 2). 149 cancer cases with 1654.9 per 100,000 person-years incidence and 64 cancer cases with 1139.1 per 100,000 person-years incidence were observed in male and female liver recipients (data not shown). The three top-most commonly incident cancers were liver cancer (n = 43), stomach cancer (n = 15), and non-Hodgkin’s lymphoma (n = 13) in males and liver cancer (n = 11), non-Hodgkin’s lymphoma (n = 10), and breast cancer (n = 9) in females.

Risk of all cancer development was two or three-fold higher than general population (SIR = 3.43, 95% CI = 2.88–3.99 in males, SIR = 2.30, 95% CI = 1.74–2.86 in females). In males, although the SIR of Kaposi’s sarcoma was the highest, the statistical significance was not observed due to small number of cases (1 case). The SIR of non-Hodgkin’s lymphoma was the significantly higher with SIR of 15.33 (95% CI = 7.00–23.66) followed by leukemia and skin cancer (SIR: 14.08 and 7.21). In addition, SIRs of liver, colorectal, and male genital cancer were significantly increased in males. In females, the SIR of non-Hodgkin’s lymphoma was significantly higher with SIR of 26.97 (95% CI = 10.26–43.69) followed by liver cancer (SIR = 11.28, 95% CI = 4.61–17.95). Other types of cancer did not show significant increment.

### Cancer incidence in kidney and liver transplant recipients by age at transplantation

We found an increased risk of overall cancer in younger recipients (aged 0–19 at transplantation) with especially higher SIR than other age groups (Table 3). The SIR of cancer in male kidney recipients aged 0–19 was 37.87 (95% CI = 4.68–71.07) then decreased as age group increased. The SIRs in female kidney recipients aged 0–19, 20–39, 40–64, and 65 or more were 104.35 (95% CI = 32.04–176.66), 4.31 (95% CI = 2.88–5.73), 2.28 (95% CI = 1.88–2.67), and 1.42 (95% CI = 0.04–2.80), respectively. In male liver recipients, the SIRs of overall cancer were 41.44 (95% CI = 0.83–82.05), 5.50 (95% CI = 0.11–10.89), and 2.27 (95% CI = 1.08–3.46) in aged 0–19, 20–39, 40–64, and 65 or more. Corresponding values for female liver recipients were 82.19 (95% CI = 31.25–133.14), 5.08 (95% CI = 1.32–8.85), 1.87 (95% CI = 1.29–2.45), and 1.81 (95% CI = 0.47–3.15), respectively.

**Table 3:** Standardized incidence ratio of all cancer and non-Hodgkin lymphoma in kidney and liver transplant recipients by age at transplantation.

Cancer site	ICD-10	Organ	Male				Female			
		Age group*	O	E	SIR	95% CI	O	E	SIR	95% CI
All cancers	C00-C96	Kidney								
		0–19	5	0.1	37.87	4.68–71.07	8	0.1	104.35	32.04–176.66
		20–39	38	3.9	9.86	6.73–13.00	35	8.1	4.31	2.88–5.73
		40–64	217	70.9	3.06	2.65–3.47	129	56.7	2.28	1.88–2.67
		65–	29	14.5	2.00	1.27–2.72	4	2.8	1.42	0.03–2.80
		Liver								
		0–19	4	0.1	41.44	0.83–82.05	10	0.1	82.19	31.25–133.14
		20–39	4	0.7	5.50	0.11–10.89	7	1.4	5.08	1.32–8.85
		40–64	127	36.4	3.49	2.88–4.10	40	21.4	1.87	1.29–2.45
		65–	14	6.2	2.27	1.08–3.46	7	3.9	1.81	0.47–3.15
non-Hodgkin lymphoma	C82-C86, C96	Kidney								
		0–19	4	0.0	218.20	4.37–432.03	4	0.0	643.92	12.89–1274.94
		20–39	7	0.2	39.39	10.21–68.56	5	0.1	47.97	5.92–90.02
		40–64	11	1.4	8.02	3.28–12.76	4	0.6	6.16	0.12–12.20
		65−	0	0.2	—	—	0	0.1	—	—
		Liver								
		0–19	4	0.0	298.46	5.97–590.95	6	0.0	608.63	121.63–1095.64
		20–39	1	0.0	29.82	0–88.26	0	0.0	—	—
		40–64	8	0.7	11.36	3.49–19.24	3	0.2	12.24	0–26.12
		65−	0	0.1	—	—	1	0.1	12.65	0–37.46

*Age at receiving transplantation.

When stratified by cancer type, due to limited number of incidence by cancer type in each age group, most of the cancer types did not show the significant results (data not shown). However, the SIRs of non-Hodgkin’s lymphoma were prominent in those aged 0–19 with 200 or more value (SIR = 218.20 [95% CI = 4.37–432.03] in male kidney recipients, 643.92 [95% CI = 12.89–1274.94] in female kidney recipients, 298.46 [95% CI = 5.97–590.95] in male liver recipients, and 608.63 [95% CI = 121.63–1095.64] in female liver recipients) which was much higher than other age groups.

## Discussion

To the best of the authors’ knowledge, this is the first nationwide population based study to investigate post-transplantation *de novo* cancer development in Korea. We observed a two to three-fold increased risk of overall cancer in kidney and liver transplant recipients, in comparison with general population. Especially, the elevated cancer risk was more prominent in younger individuals. The types of cancer showing increased risk were various including infection related and unrelated cancers. Among kidney recipients, cancers in thyroid and endocrine glands and kidney were the most commonly observed, and for liver recipients, liver cancer was the most common malignancy after transplantation. When stratified by sex, cancers in kidney, stomach, thyroid and endocrine gland, and skin were most commonly developed in male kidney recipients, while cancers in thyroid and endocrine gland, breast, kidney, and genital were most common in female kidney recipients. Among liver recipients, liver cancer, stomach cancer, and non-Hodgkin’s lymphoma were most common in men and liver cancer, non-Hodgkin’s lymphoma, and breast cancer were most common in female.

In comparison with general population, despite of statistical significance in only male kidney recipients due to small number of incident cases in other groups, the risk of Kaposi sarcoma in kidney recipients was the most prominent with SIR of 100 to 300. Kaposi sarcoma is one of the important infection related cancer. Many studies have suggested that immunosuppression therapy after transplantation could increase infection with viruses such as human herpes virus which plays an important role in the etiology followed by increased incidence of Kaposi sarcoma, especially in kidney recipients^23^. In previous studies targeting Western populations, the excess risk of Kaposi sarcoma incidence was prominent with the SIR value of 40 or more^8,9^, reached to more than 100 ^19^. Studies in Asian populations did not show the SIR of Kaposi sarcoma due to unavailable incidence rate in general population^14^ or lower SIR value^16,24^ than our results. The incidence of Kaposi sarcoma is lower in the general population with Asian ethnicity than other populations^25^, especially in Korean population^26^, and it would be the reason of the extremely high SIR despite the small number of cases in transplantation recipients. In female kidney recipients, even with one incident case, the SIR of Kaposi sarcoma was more than 300.

Non-Hodgkin’s lymphoma, whose risks were greatly increased in both kidney and liver recipients, is also a well-known major post-transplant malignancy. Non-Hodgkin’s lymphoma is one extreme of Epstein–Barr virus infection-related proliferative disease, a major part of post-transplant lymphoproliferative disorder. Although half of the post-transplant lymphoproliferative disorders are Epstein–Barr virus negative, infection with viruses and immunosuppression are suspected to be causes for them^27^. Also, the increased cancer risks in male/female genital organs are suspected to be infection related such as human papilloma virus.

Risks of cancers in bone, articular cartilage, skin, mesothelium, and soft tissue were also increased in male kidney or liver recipients. In this study we grouped cancers as ICD-10 code C40-C49, except Kaposi sarcoma (C46) due to small number of each type of cancer, and skin cancer accounted for most of them (31 of 35 in kidney recipients and 7 of 10 in liver recipients). Skin cancer after transplantation is considered one of the main concerns in Western countries with most common post-transplantation malignancy^8–10,19^, but in Asian countries, studies showed various risks of skin cancer including increased risk^13,16^, non-significantly increment^14^, or not considered due to limited number of cases in general population^24^. For the cause of skin cancer after transplantation, not only viral infection whose evidence was inconsistent^28,29^ but also UV light exposure and other carcinogens are suggested^9^. One study proposed age as the most important risk factor of skin cancer after transplantation and would be a proxy for association with infection unrelated causes^17^. A previous study conducted in Korea showed that among non-melanoma skin cancers after kidney transplantation, squamous cell carcinoma was most common^24^, as is in Western countries^9^, but we could not identify subtypes of skin cancer. A statistically insignificant increased risk in female recipients in this study population might be caused by a small number of malignant cases.

A leukemia risk after solid organ^8,30^ or liver transplantation^16^ was also observed in this study, and was especially higher in younger individuals (data not shown). Immune dysfunctions or immune suppression are suggested to be an etiology of increased risk^30^. Although acute myeloid leukemia was mostly considered type of hematopoietic malignant in previous studies^31,32^, studies showed that most types of leukemia increased after transplantation^8,30^. In this study, only male kidney or liver recipients showed an increased risk of leukemia and when stratified by age group, due to small number of incident cases in younger age group (1 case), only male recipients aged 40–64 showed significantly higher SIR. In addition, it was impossible to observe risk in female recipients due to negligible number of leukemia cases (0 or 1).

Incidence of cancers in bladder (female) and kidney (both genders) in kidney recipients, as well as liver cancer in liver transplant recipients were higher than the general population which were comparable with previous studies^2,8–10,13–16^. The elevated cancer risk at the respective transplantation site would be related to the indication of transplantation. Dialysis periods, existing hepatitis, and alcohol intake tendencies are thought to have affected the results. In addition, cigarette smoking is an established risk factor of lung cancer as well as chronic kidney disease^33^ and the smoking rate of patients transplanted for alcoholic liver diseases were higher^34^. Thus, a shared risk factor for both diseases would explain the increased risk of lung cancer in male kidney and liver recipients. Previous studies showed that liver recipients due to alcoholic liver disease had higher risk of *de novo* cancer incidence, especially smoking related cancer including cancer in lung, larynx, lip-mouth-pharynx, and esophagus^35,36^. Higher smoking prevalence in men^37^ may explain higher lung cancer risk only in male kidney recipients.

The excess risk of cancers in thyroid and endocrine glands, stomach, breast in kidney recipients and colorectal cancer in male liver recipients, which also has been demonstrated previously, may result from increased surveillance. In Korea, screening for stomach, colorectal, and breast cancer are included in the national cancer screening program^38^ and screening for thyroid cancer is widely available^39^. However, considering the differently increased cancer of these types between kidney and liver recipients, a true biologic effect cannot be excluded. Studies suggested that the main explanation for the different cancer risk by transplanted organ is variation in the type of immunosuppression or its intensity^10,19,40^.

Studies on post-transplantation malignancy in pediatric transplant recipients were scarce, but they reported the increased cancer risk including overall cancer, non-Hodgkin’s lymphoma, myeloma, Hodgkin’s lymphoma, and leukemia^41,42^. We found an increased risk of overall cancer in younger recipients (aged 0–19 at receiving transplantation) with higher SIR than recipients aged 40 or older. However, due to limited number of incidence in this age group, statistically significantly increased SIR could be observed only for non-Hodgkin’s lymphoma. The increased risk of non-Hodgkin’s lymphoma in younger solid organ recipients has been observed consistently in Western countries^18–20,41^. Considering the suggested factors associated with non-Hodgkin’s lymphoma including infection and immunosuppression^27^ and longer expected years with transplanted organ in younger recipients, antiviral prophylaxis and monitoring of EBV viral load is recommended for young high‐risk recipients^43^.

Potential limitations of this study need to be mentioned. First, kidney and liver transplant recipients and cancer incidence were identified through medical insurance code, ICD-10 code, and catastrophic illness registry in HIRA claim data, with potential misclassification. However, kidney and liver transplantation is a major medical treatment and reimbursed from the National Health Insurance. Applying catastrophic illness registry to define cancer is related with reimbursement of copayment, requiring relevant clinical information for approval by the insurance administration. Thus definition of recipients and cancer would be valid. Second, although we standardized age and stratified by sex to eliminate the effect of differences on cancer incidence between recipients and general population, other risk factors of cancer such as smoking, drinking, or sunlight exposure were not considered due to a lack of information. Despite the distinct etiology of liver and kidney transplantation in Korea, where hepatitis B infections and glomerulonephritis are the most common cause of liver and kidney transplantation, respectively^44–46^, the *de novo* cancer incidence could not be compared according to the cause of transplantation due to the methodological issue in indirect standardization^47^. In addition, a maximum eight-year study follow-up period might not be enough for all possible cancer development, but our results would be comparable for major types of cancer incidence after transplantation with previous studies. Although *de novo* cancers, especially infection-related cancers, developed after transplantation are known to be largely induced by immunosuppression therapy, we did not have the exact data on individuals’ response to immunosuppression therapy such as human herpes virus or Epstein–Barr virus infections. Additionally, we did not have the data on several blood tests that can predict the level of immunosuppression, such as white blood cell count, due to the limitations of claim data. Thus, the causal pathway in the process of transplantation – immunosuppression therapy – infection – infection related *de novo* cancer could not be clarified. Since 2000, a common protocol of immunosuppressant therapy after transplantation has been applied in Korea, which generally includes the use of steroids, calcineurin inhibitor drugs, and mycophenolate mofetil as an initial maintenance treatment; steroids are tapered off and withdrawn after several months in both liver and kidney recipients. Thus, despite the possible differences in the actual administration between different medical centers, the variations in the immunosuppression regimens for the recipients would not be significant. In addition, the protocols include the administration of several immunosuppressants together or gradually. Since we assessed the incidence ratio, it was difficult to identify the individual effect of each immunosuppressant on *de novo* cancer. Despite these limitations, this study included almost the entire population of Korea with representativeness and first report targeting kidney and liver recipients in Korea.

In conclusion, the incidence of cancer in kidney and liver transplant recipients was two to three-fold higher than general population. The commonly incident types of cancer were different from the general population with higher SIRs for non-Hodgkin’s lymphoma, Kaposi sarcoma (both sex), skin cancer, leukemia (male), and respective transplantation site which were comparable with previous studies in Western population as well as screening related cancers. Especially, an increased risk for overall cancer and non-Hodgkin’s lymphoma in young recipients was notable. Better knowledge of the specificities of kidney and liver recipients with *de novo* cancer is required to improve care in solid organ recipients. Considering that screening for some commonly developed cancers in organ recipients would be highly beneficial^36^, surveillance protocol for cancers with high risk would be considered for these population. This study showed important considerations for strategies in cancer screening and surveillance in kidney and liver recipients for improvement of outcomes. Further studies are needed to identify the risk factors for commonly developed cancers in solid organ recipients, differences in cancer incidence according to the underlying etiology of transplantation adjusted for age and sex, and the effects of *de novo* cancer on the prognosis or survival in recipients.

## Data Availability

Data is available on request to the Healthcare Bigdata Hub, Health Insurance Review & Assessment, Korea.

## References

[1] Asch WS, Perazella MA (2016). Cancer and Mortality in Solid-Organ Transplantation: Preventable or Inevitable?. Am J Kidney Dis.

[2] Global Observatory on Donation and Transplantation. World Health Organization Collaborating Center on Donation and Transplantation. <Available at: http://www.transplant-observatory.org/>. Last accessed 16 Dec 2018.

[3] Hart A (2018). OPTN/SRTR 2016 Annual Data Report: Kidney. Am J Transplant.

[4] Kim WR (2018). OPTN/SRTR 2016 Annual Data Report: Liver. Am J Transplant.

[5] Doycheva I, Amer S, Watt KD (2016). De Novo Malignancies After Transplantation: Risk and Surveillance Strategies. Med Clin North Am.

[6] Watt KD (2009). Long-term probability of and mortality from de novo malignancy after liver transplantation. Gastroenterology.

[7] McCaughan JA, Courtney AE (2015). The clinical course of kidney transplant recipients after 20 years of graft function. Am J Transplant.

[8] Engels EA (2011). Spectrum of cancer risk among US solid organ transplant recipients. JAMA.

[9] Krynitz B (2013). Risk of skin cancer and other malignancies in kidney, liver, heart and lung transplant recipients 1970 to 2008–a Swedish population-based study. Int J Cancer.

[10] Collett D, Mumford L, Banner NR, Neuberger J, Watson C (2010). Comparison of the incidence of malignancy in recipients of different types of organ: a UK Registry audit. Am J Transplant.

[11] Acuna SA (2017). Cancer recurrence after solid organ transplantation: A systematic review and meta-analysis. Transplant Rev (Orlando).

[12] Grulich AE, van Leeuwen MT, Falster MO, Vajdic CM (2007). Incidence of cancers in people with HIV/AIDS compared with immunosuppressed transplant recipients: a meta-analysis. Lancet.

[13] Cheung CY (2012). Malignancies after kidney transplantation: Hong Kong renal registry. Am J Transplant.

[14] Lee KF (2016). Cancer Incidence among Heart, Kidney, and Liver Transplant Recipients in Taiwan. PLoS One.

[15] Li, W. H. *et al*. Malignancies after renal transplantation in Taiwan: a nationwide population-based study. *Nephrol Dial Transplant* **27**, 833–839, 10.18632/oncotarget.13124, 10.1093/ndt/gfr277 (2012). 10.1093/ndt/gfr277 21633099

[16] Tsai HI (2017). De novo malignancy in organ transplant recipients in Taiwan: a nationwide cohort population study. Oncotarget.

[17] Tsai YF (2016). Nationwide population-based study reveals increased malignancy risk in taiwanese liver transplant recipients. Oncotarget.

[18] Jiang Y (2008). Liver transplantation and subsequent risk of cancer: findings from a Canadian cohort study. Liver Transpl.

[19] Na R (2013). Comparison of de novo cancer incidence in Australian liver, heart and lung transplant recipients. Am J Transplant.

[20] Villeneuve PJ (2007). Cancer incidence among Canadian kidney transplant recipients. Am J Transplant.

[21] Shin A (2011). Population attributable fraction of infection-related cancers in Korea. Ann Oncol.

[22] Kim L, Kim JA, Kim S (2014). A guide for the utilization of Health Insurance Review and Assessment Service National Patient Samples. Epidemiol Health.

[23] Frances C (2009). The impact of preexisting or acquired Kaposi sarcoma herpesvirus infection in kidney transplant recipients on morbidity and survival. Am J Transplant.

[24] Kim JH, Kim SO, Han DJ, Park SK (2014). Post-transplant malignancy: a burdensome complication in renal allograft recipients in Korea. Clin Transplant.

[25] Liu Z (2018). The world-wide incidence of Kaposi’s sarcoma in the HIV/AIDS era. HIV Med.

[26] de Sanjose S (2009). Geographic variation in the prevalence of Kaposi sarcoma-associated herpesvirus and risk factors for transmission. J Infect Dis.

[27] Dierickx D, Habermann TM (2018). Post-Transplantation Lymphoproliferative Disorders in Adults. N Engl J Med.

[28] Dubina M, Goldenberg G (2009). Viral-associated nonmelanoma skin cancers: a review. Am J Dermatopathol.

[29] Schulz TF (2009). Cancer and viral infections in immunocompromised individuals. Int J Cancer.

[30] Morton LM (2014). Risk of myeloid neoplasms after solid organ transplantation. Leukemia.

[31] Rashidi A, Fisher SI (2014). Acute myeloid leukemia following solid organ transplantation: entity or novelty?. Eur J Haematol.

[32] Morton, L. M. *et al*. Risk of Acute Myeloid Luekemia Among Solid Organ Transplant Recipients. *Blood* **118**, 2559, 10.1182/blood.V118.21.2559.2559 (2011).

[33] Xia J (2017). Cigarette smoking and chronic kidney disease in the general population: a systematic review and meta-analysis of prospective cohort studies. Nephrol Dial Transplant.

[34] Jimenez C (2007). Incidence and risk factors for the development of lung tumors after liver transplantation. Transpl Int.

[35] Seree O (2018). Longterm Risk of Solid Organ De Novo Malignancies After Liver Transplantation: A French National Study on 11,226 Patients. Liver Transpl.

[36] Renaud L (2018). De Novo Malignancies Screening After Liver Transplantation for Alcoholic Liver Disease: A Comparative Opportunistic Study. Liver Transpl.

[37] Chung W, Lim S, Lee S (2010). Factors influencing gender differences in smoking and their separate contributions: evidence from South Korea. Soc Sci Med.

[38] Suh M (2017). Trends in Participation Rates for the National Cancer Screening Program in Korea, 2002-2012. Cancer Res Treat.

[39] Park S (2016). Association between screening and the thyroid cancer “epidemic” in South Korea: evidence from a nationwide study. BMJ.

[40] Euvrard S (1995). Comparative epidemiologic study of premalignant and malignant epithelial cutaneous lesions developing after kidney and heart transplantation. J Am Acad Dermatol.

[41] Yanik, E. L. *et al*. Cancer Risk After Pediatric Solid Organ Transplantation. *Pediatrics* **139** (2017). 10.1542/peds.2016-3893 PMC5404730 28557749

[42] Kitchlu, A. *et al*. Elevated Risk of Cancer Following Solid Organ Transplant in Childhood: A Population-based Cohort Study. *Transplantation*, 10.1097/tp.0000000000002378 (2018). 10.1097/TP.0000000000002378 30048393

[43] Lee TC (2005). Quantitative EBV viral loads and immunosuppression alterations can decrease PTLD incidence in pediatric liver transplant recipients. Am J Transplant.

[44] Lee SG (2005). [Current status of liver transplantation in Korea]. Korean J Gastroenterol.

[45] Kwak BJ (2018). Clinical outcome of 1,000 consecutive cases of liver transplantation: a single center experience. Ann Surg Treat Res.

[46] Ahn C (2014). Initial Report of the Korean Organ Transplant Registry: The First Report of National Kidney Transplantation Data. Transplantation Proceedings.

[47] Julious SA, Nicholl J, George S (2001). Why do we continue to use standardized mortality ratios for small area comparisons?. J Public Health Med.

